# Contemporary Management and Attainment of Cholesterol Targets for Patients with Dyslipidemia in China

**DOI:** 10.1371/journal.pone.0047681

**Published:** 2013-04-09

**Authors:** Fei Gao, Yu Jie Zhou, Da Yi Hu, Ying Xin Zhao, Yu Yang Liu, Zhi Jian Wang, Shi Wei Yang, Xiao Li Liu

**Affiliations:** 1 Department of Cardiology, An Zhen Hospital, Capital Medical University, Beijing, China; 2 Heart Centre, Peking University People's Hospital, Beijing, China; Universidad Peruana Cayetano Heredia, Peru

## Abstract

**Aims:**

It is well-established that lipid disorder is an important cardiovascular risk factor, and failure to reach optimal lipid levels significantly contributes to the residual cardiovascular risks. However, limited information is available on the management and the attainment of recommended cholesterol targets in real-world practice in China.

**Methods and Results:**

A nationally representative sample of 12,040 patients with dyslipidemia from 19 provinces and 84 hospitals across China were consecutively enrolled in this survey. Risk stratification and individual cholesterol target was established for all participants. This survey identified a high-risk cohort, with over 50% of patients had hypertension, 37.5% had coronary artery disease, and more than 30% had peripheral artery disease. Thirty-nine percent of all participants received lipid lowering medications. And the majority of them (94.5%) had statins (42.5% with atorvastatin, 29.0% with simvastatin, and 15.2% with rosuvastatin). However, the overall attainment for low-density lipoprotein cholesterol (LDL-C) target is low (25.8%), especially, in female (22.2%), and in patients with increased body mass index (BMI) (38.3% for BMI<18.5, 28.1% for BMI 18.5–24.9, 26.0% for BMI 25.0–29.9, and 17.4% for BMI≥30, P<0.0001). Subgroup analysis also showed the attainment is significantly lower in patients who were stratified into high (19.9%) and very high (21.1%) risk category. In logistic regression analysis, eight factors (BMI, gender, coronary artery disease, systolic and diastolic blood pressure, hypertension, family history of premature coronary artery disease and current smoking) were identified as independent predictors of LDL-C attainment.

**Conclusions:**

Despite the proven benefits of lipid-lowering therapies, current management of dyslipidemia continues to be unsatisfied. A considerable proportion of patients failed to achieve guideline-recommended targets in China, and this apparent treatment gap was more pronounced among patients with increased BMI, higher risk stratification and women.

## Introduction

The strong link between dyslipidemia and the risk of future cardiovascular events is well understood. [1]–[7] Previous studies have also demonstrated an important reduction in cardiovascular morbidity and mortality with low-density lipoprotein cholesterol (LDL-C) – lowering therapies. [8]–[12] Furthermore, the benefits of treatment appear more pronounced among patients with established cardiovascular disease or in those at high risk of developing cardiovascular disease. Therefore, the National Cholesterol Education Program (NCEP) Adult Treatment Panel III (ATP III) guidelines highlight the importance of the LDL-C as the primary therapeutic target and recommend individualized treatment goals tailored to the estimated cardiovascular risk [1], [2]. And failure to reach recommended lipid goals is associated with increased risk of cardiovascular events [13]–[15]. However, despite the well-established efficacy of lipid-modifying therapy, limited data are available on guideline attainment in ambulatory patients with dyslipidemia in China.

We herein aimed to investigate (1) the current attainment of the recommended LDL-C targets among patients with dyslipidemia in Chinese outpatient clinic, (2) sex differences in attainment of guideline-recommended targets for patients with dyslipidemia, and (3) whether attainment of the recommended LDL-C targets are similar in ambulatory patients stratified by different risk category.

## Methods

The Reality China survey is a multi-centre cross-sectional study involving 84 centers in 19 provinces in China. The sampling process was stratified according to geographic region (north, east, south central, northwest, and southwest China), and degree of urbanization (large cities [Beijing, Shanghai, and provincial capitals] and midsize cities) (Supplement S1). The first two stages of sampling, in which provinces were selected from geographic regions and cities were selected from provinces, were not random. In the next stage (the stage in which hospital or centers were selected from cities or townships), the sampling was random. Each center was asked to recruit at least 20 patients. Consecutive patients at an age ≥20 years were screened for a previously or newly diagnosed dyslipidemia when visiting the outpatient clinics. All patients were assessed and treated at the discretion of their physicians in charge according to the usual institutional practice. A standard case record form (CRF) was administered by trained staff to obtain information on demography, conventional risk factors for cardiovascular disease, family history of premature coronary artery disease (CAD), medical history and treatment. All study investigators and staff members completed a training program that they were given detailed instructions concerning the administration of the study CRF. The following data were obtained on enrolment day: weight, height, fasting plasma glucose, systolic and diastolic blood pressure and fasting blood lipids (total-, LDL- and high-density lipoprotein-cholesterol, triglycerides). Estimated glomerular filtration rate (eGFR) was calculated by modified MDRD equation [16] And if eGFR <60 ml/min/1.73 m [2], patients were defined as chronic kidney disease. If patient had more than one clinic visit during the study period, data from the index encounter were used to minimize overrepresentation by patients with multiple visits.

The assessment of risk factors was based on the NCEP ATP-III guidelines update [2] and included: age (≥45 years if male or ≥55 years if female), hypertension, low HDL cholesterol (<40 mg/dL), cigarette smoking, family history of premature CAD (first degree relatives before the age of 55 in men and 65 years in women). CAD risk equivalents included: stroke or transient ischemic attack, peripheral arterial disease, aortic abdominal aneurysms, diabetes, multiple risk factors and 10-year Framingham risk >20%. Risk assessment for determining 10-year CAD risk was carried out according to Framingham risk scoring. [1] Diabetes was defined according to medical records or at least two fasting glucose measurements ≥7.0 mmol/L during recruitment. Personal history of CAD and CAD risk equivalents was established according to patients' medical records. Participants were stratified into risk categories according to the cumulative number of risk factors and the Framingham risk score, as reported by the NCEP ATP-III guidelines. The following risk categories that modify LDL-C goals were generated: less than 160 mg/dL (4.14 mmol/L) if low risk (0–1 risk factor), less than 130 mg/dL (3.37 mmol/L) if moderate risk (≥2 risk factors and Framingham risk score 10–20%), less than 100 mg/dL (2.59 mmol/L) if high risk (CAD or CAD risk equivalents) and less than 70 mg/dL (1.81 mmol/L) for very high-risk patients (those who have had a recent heart attack, or have cardiovascular disease combined with diabetes). [2] All participants were investigated on whether they attained individualized LDL-C targets.

The study protocol was approved by the certified central Ethics Committee of An Zhen Hospital, Capital Medical University, Beijing, China. And written informed consent was obtained from all participants in this study.

### Statistics

All case record form data were entered into two Epidata 3.02 databases by different people. The two databases were compared, and any necessary corrections were discussed and reassessed. The data were analyzed using SPSS statistical software 13.0. Categorical data are presented as frequencies (percentages), and the differences were compared by the *x* ^2^ test; continuous data are presented as mean value +− SD and the differences between groups were compared by the t test. The predictors of LDL-C target attainment failure were investigated by multivariable logistic regression analysis adjusted for baseline characteristics and all potential confounding factors, including demographic factors (age, BMI [categorical variable] and gender), clinical characteristics (diabetes, current smoking, hypertension, CAD, stroke/TIA, peripheral artery disease, chronic kidney disease, aortic abdominal aneurysms and family history of premature CAD), physical examination (systolic and diastolic blood pressure) and treatment status (administration of lipid lowering medication, adverse reaction events related to lipid lowering therapy). All p values were 2-sided, and a p value of <0.05 was considered statistically significant.

## Results

Enrollment of patients was initiated in March, 2011 and continued until December, 2011 with starting time varying between centers. Each center collected patients during 2–6 weeks. A total of 12,040 eligible patients with dyslipidemia were enrolled and 1082 subjects were excluded due to protocol violations or missing data. The geographical distribution of participants was relatively well balanced between the north (29%), east (33%), south central (19%), northwest (10%), and southwest (10%) regions of China. Sixty-nine percent of the centers were Tertiary University hospitals.

### Risk characteristics of the cohort

The baseline characteristics demonstrated a high risk cohort, with over 50% of patients had concomitant hypertension, 37.5% had coronary artery disease, 17.5% had diabetes and more than 30% had peripheral artery disease (Table 1). In the overall cohort, there were 28.1%, 23.8%, 25.0% and 23.1% of patients had optimal (LDL-C<100 mg/dl), above optimal (130 mg/dl >LDL-C≥100 mg/dl), borderline high (160 mg/dl >LDL-C≥130 mg/dl) and high LDL-C levels (LDL-C≥160 mg/dl) based on the ATP III guidelines. Patients were further divided into different risk categories according to their individual risk factors: 1926 patients (16%) in very high risk category, 5177 patients (43%) in high risk category, 2528 patients (21%) in moderate risk category, and 2408 patients (20%) in low risk category.

**Table 1:** Characteristics.

Characteristics	Total (N = 12040)	Male (N = 6860)	Female (N = 5180)
**Age (years)**	61.7±12.5	60.9±13.0	62.8±11.9*
**BMI (kg/m^2^)**	24.3±3.5	24.4±3.4	24.1±3.7*
**Diabetes (%)**	17.5	17.4	17.6
**Hypertension (%)**	51.9	52.1	51.6
**Smoking (%) Never/former/current**	67.9/9.9/22.2	46.8/16.5/36.6	95.8/1.1/3.1*
**CAD (%)**	37.5	41.0	32.9*
**Stroke/TIA (%)**	12.3	12.6	12.0
**Peripheral artery disease (%)**	31.2	30.9	31.7
**Chronic kidney disease (%)**	3.3	3.4	3.3
**Aortic abdominal aneurysms (%)**	2.3	2.9	1.6
**Family history of premature CAD (%)**	5.7	5.7	5.6
**PCI**	22.3	25.3	18.3*
**CABG**	2.1	2.4	1.7
**SBP (mmHg)**	135.1±18.2	134.9±18.0	135.3±18.4
**DBP (mmHg)**	82.7±12.4	83.0±12.3	82.1±12.4*
**Total cholesterol (mg/dl)**	199.9±58.7	194.7±59.0	206.9±57.5*
**LDL- C (mg/dl)**	128.3±46.3	124.7±44.9	133.2±47.7*
**HDL- C (mg/dl)**	49.5±26.9	47.7±27.5	51.8±25.9*
**Triglyceride (mg/dl)**	194.6±133.5	196.2±140.7	192.5±123.2

* **P<0.0001 for comparison between female and male.**

BMI: body mass index; TIA: transient ischemic attack; CAD: coronary artery disease; PCI: percutaneous coronary intervention; CABG: coronary artery bypass graft; SBP: systolic blood pressure; DBP: diastolic blood pressure; LDL-C: low-density lipoprotein cholesterol; HDL-C: high-density lipoprotein cholesterol.

**Figure 1:**
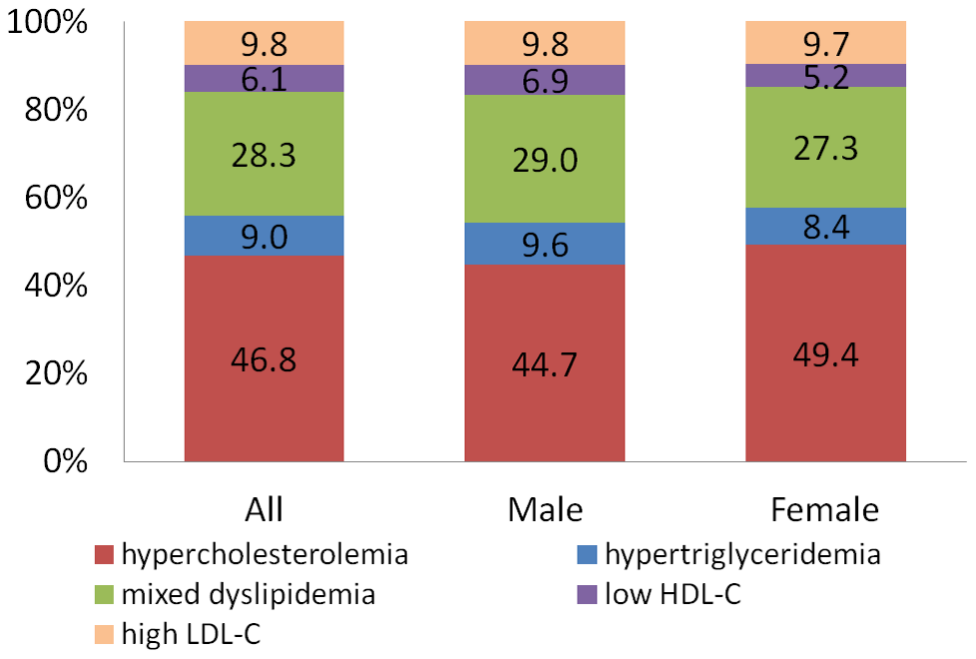
The proportion of patients with individual or mixed lipid disorders.

Notably, there were statistically significant differences between men and women in age, body mass index (BMI), smoking status, coronary artery disease and percutaneous coronary intervention proportions. Furthermore, the mean levels of total cholesterol, LDL- C and HDL- C were also significantly higher in female, whereas the diastolic blood pressure was lower in comparison with male (Table 1). The proportion of patients with individual or mixed lipid abnormalities was shown in Figure 1. Female had a higher prevalence of hypercholesterolemia. Conversely, a higher proportion of hypertriglyceridemia, low HDL-cholesterol and mixed lipid disorders were observed in male than in female (Figure 1).

### Lipid modification therapy

In this cohort, 39.3% of all participants received lipid lowering medications before study enrollment. In addition, even for patients in high or very high risk group, the prescription rate was only 42.1% and 46.5%, respectively. And the prescription of lipid lowering medications was strikingly low among patients enrolled from non-tertiary hospitals (31.1%). In patients with lipid lowering medications, the majority of them (94.5%) had statins (42.5% with atorvastatin, 29.0% with simvastatin, and 15.2% with rosuvastatin). The adverse reaction events were rare and did not differ from man and women (Table 2). And there were 41.3% of patients in the overall cohort had received blood lipid tests, and 30.8% of patients had creatine kinase and liver enzyme monitoring within three months before enrollment (Table 2).

**Table 2:** Lipid lowering therapy.

	Total (N = 12040)	Male (N = 6860)	Female (N = 5180)
**Prior lipid lowering therapy (%)**	39.3	39.5	39.0
** Statins (%)**	37.1	37.5	36.5
** Niacin (%)**	1.5	1.4	1.7
** Fibrates (%)**	6.0	6.2	5.8
**Monitoring of blood lipid 3 months prior to enrollment (%)**	41.3	40.7	42.2
**Monitoring of creatine kinase and Liver enzyme 3 months prior to enrollment (%)**	30.8	30.9	30.6
**Adverse effect (%)**	1.5	1.5	1.5
** Myalgia (%)**	0.3	0.3	0.3
** Elevation of Liver enzyme (%)**	0.4	0.4	0.4
** Elevation of creatine kinase (%)**	0.04	0.06	0.02

### Attainment of recommended cholesterol targets

**Figure 2:**
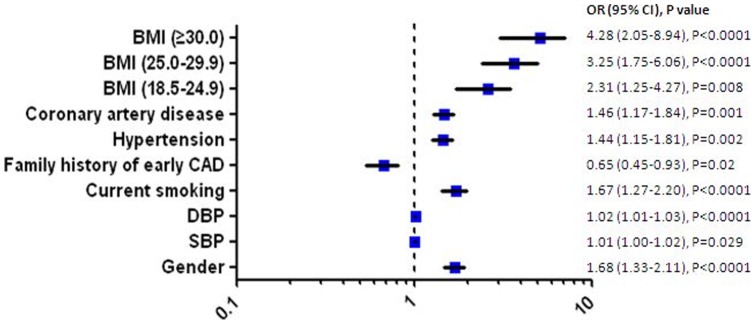
Independent predictors of failure to reach recommended LDL-Cholesterol targets.

After establishing individualized LDL-C target, the overall attainment for LDL-C target was low in this survey (25.8%), especially, in female, and in patients with increased BMI as well. The attainment was significantly lower for patients in high (19.9%) and very high (21.1%) risk category (Table 3). And among patients with lipid lowering therapy, a significantly increased LDL-C attainment rate was observed in patients taking rosuvastatin (Table 3). In multivariate logistic regression analysis, eight factors (BMI, gender, coronary artery disease, systolic and diastolic blood pressure, hypertension, family history of premature CAD and current smoking) were identified as independent predictors for failure to reach recommended LDL-Cholesterol targets (Figure 2).

**Table 3:** Attainment of recommended cholesterol targets in each subgroup.

	Number of patients in each subgroup	LDL-C attainment rate (%)	P value for difference among subgroup
**Gender**			P<0.0001
Male	6860	28.5	
Female	5180	22.2	
**BMI (Kg/m^2^)**			P<0.0001
<18.5	300	38.3	
18.5–24.9	6252	28.1	
25.0–29.9	3676	26.0	
≥30.0	533	17.4	
**Age group (years)**			P<0.0001
20–29	69	30.4	
30–39	431	24.6	
40–49	1505	23.8	
50–59	3176	23.3	
60–69	3386	24.3	
70–79	2518	29.7	
≥80	955	32.5	
**Risk stratification**			P<0.0001
Low	2408	38.1	
Moderate	2528	29.7	
High	5177	19.9	
Very high	1926	21.1	
**Centers**			P<0.0001
Tertiary	8668	28.0	
Non-tertiary	3372	20.1	
**Statin treatment group**			P<0.0001
Simvastatin	1376	36.2	
Atorvastatin	2011	34.9	
Rosuvastatin	717	51.1	
Others	628	40.0	

BMI: body mass index.

## Discussion

In this large, multi-centre, cross-sectional cohort study of 12040 high-risk ambulatory patients with lipid disorders, we demonstrated that less than 40% of patients received lipid lowering medications and only about a quarter achieved the target LDL-C levels. In particular, patients in high risk category, with increased BMI and female were less likely to meet the recommended LDL-C targets.

Although sufficient clinical studies have shown the definitive benefits of lipid-lowering therapy and LDL-C attainment in prevention of cardiovascular disease, [8]–[12] there is limited data on the contemporary treatment and LDL-C attainment in patients with dyslipidemia in China. Our study provides new information regarding the “real world” management in these high-risk patients across China. The finding that only about a quarter of the patients in our study attained the recommended lipid targets is significant and emphasizes important treatment gaps in China. Especially, nearly 60% of patients in this cohort were identified as high or very high risk category, which need stringent control on serum cholesterol. Early studies have indicated the LDL-C goal attainment was 38% among 4888 outpatients enrolled in the Lipid Treatment Assessment Project in the United States [17]. And more recently, Vascular Protection (VP) and Guidelines Oriented Approach to Lipid Lowering (GOALL) Registries showed their LDL attainment rate reached to 51% among 8056 outpatients with diabetes or established cardiovascular disease in Canada. [18] To our knowledge, the only existing data focused on Chinese population is REALITY-Asia study, [19] which was conducted in China, Korea, Malaysia, Singapore, Taiwan, and Thailand, retrospectively recruited 2622 patients with hypercholesterolemia newly initiated on statin monotherapy. This study documented that 48% of patients attained LDL-C targets, and the attainment rate was also significantly lower as the risk-category escalated, including 38% of those in high risk group, 62% of those in moderate risk group, and 81% of those in low risk group. Our study was conducted in a large Chinese population and we also observed the same trend. However, in our study the overall attainment was surprisingly low, only 25%, this phenomenon might be mainly explained by the low proportion of lipid lowering therapy (less than 40%) in our population. Besides, nearly 30% of participants were enrolled from community hospitals and they had even lower proportion of lipid lowering therapy (31.1%). It also contributes to the low attainment rate. And yet, we believe this result did reflect the “real world” practice and the huge gap between guidelines and daily clinical practice in China.

Another important finding of our study is that we identified several factors which would strongly affect the LDL-C attainment. First and foremost, gender is a strong predictor for attainment failure, with 22% attainment in female. This finding was consistent to VP and GOALL registry. [18], [20] In addition, our study indicated the different pattern of lipid disorder between man and woman. Specifically, female had a higher prevalence of hypercholesterolemia and a lower proportion of hypertriglyceridemia, which is similar to the study conducted by M. Pirro, et al. [21] Second important predictor for attainment failure is obesity. Patients with increased BMI had significantly lower attainment, this result concurs with V. Bhan, et al. (pos-hoc analysis of the VP and GOALL registry). [20] It reflects the difficulty in managing obese patients, who are often resistant to therapy and might require combined drug therapy, as well as lifestyle interventions. And previous observational studies also suggest patients with increased BMI had higher incidence of adverse cardiac events after percutaneous coronary intervention [22]–[24], the disparity of LDL-C attainment may be one of the reasons contributing to the worse outcome in patients with obesity. In addition, the mean BMI was lower in the China REALITY Survey population (24.3 kg/m^2^) than the VP and GOALL registry (29.3 kg/m^2^). [18] And there is growing evidence that metabolic and cardiovascular risk profiles are different in Asian and Caucasian individuals and the thresholds for diagnosing obesity are lower in Asian individuals. [25], [26] Therefore, we believe the result from our study would be more persuasive for Chinese population.

Finally, recent studies also suggest that statin could provide cardiovascular benefits beyond LDL-cholesterol lowering effect. [27] Therefore risk reduction could be achieved by statin treatment without achieving the targets due to pleiotropic effects. However, our survey also indicated that statin was only prescribed to less than 40% of overall participants. Even for high or very high risk category, the prescription rate was merely 42.1% and 46.5%, respectively. And it poses imperative demands for developing national program to raise public awareness.

### Limitations

The present study had several limitations. First, lipid measurements were not performed in a central core laboratory. However, this also reflects “real world” practice where physicians initiate or titrate therapy based on available test results. Second, we did not collect data on the duration of lipid lowering therapy or adherence to medications. Third, life-style modifications or non-pharmacological therapies were not assessed in our study. Finally, this survey is a cross-sectional study. A prospective follow-up study is required to assess the medical treatment, attainment in relation to mortality in patients with dyslipidemia.

In conclusion, despite the proven benefits of lipid-lowering therapies, current management of dyslipidemia is still suboptimal in China. A considerable proportion of patients failed to achieve guideline-recommended targets, and the treatment gap was more pronounced among patients with increased BMI, higher risk stratification and women. Our study also suggests more aggressive treatment strategies and national improvement projects should be implemented to narrow the existing gap.

### Disclosures

This study was supported by the Chinese Society of Cardiology (a federally incorporated none-profit academic research organization) and AstraZeneca. The industry sponsors had no involvement in the study conception or design; collection, analysis, and interpretation of data; in the writing of the manuscript; and in the decision to submit the manuscript for publication. The authors had full access to the data and take full responsibility for its integrity. All the authors have read and agreed to the article as written.

## Supporting Information

Supplement S1 (PPT)
