# Supplementary material for: Contemporary Management and Attainment of Cholesterol Targets for Patients with Dyslipidemia in China
Source: PLoS One. 2013 Apr 9;8(4):e47681. doi: 10.1371/journal.pone.0047681 (PMC3621908; doi:10.1371/journal.pone.0047681)
Supplement: Supplement S1 — (PPT) [file pone.0047681.s001.ppt]

## Slide 1
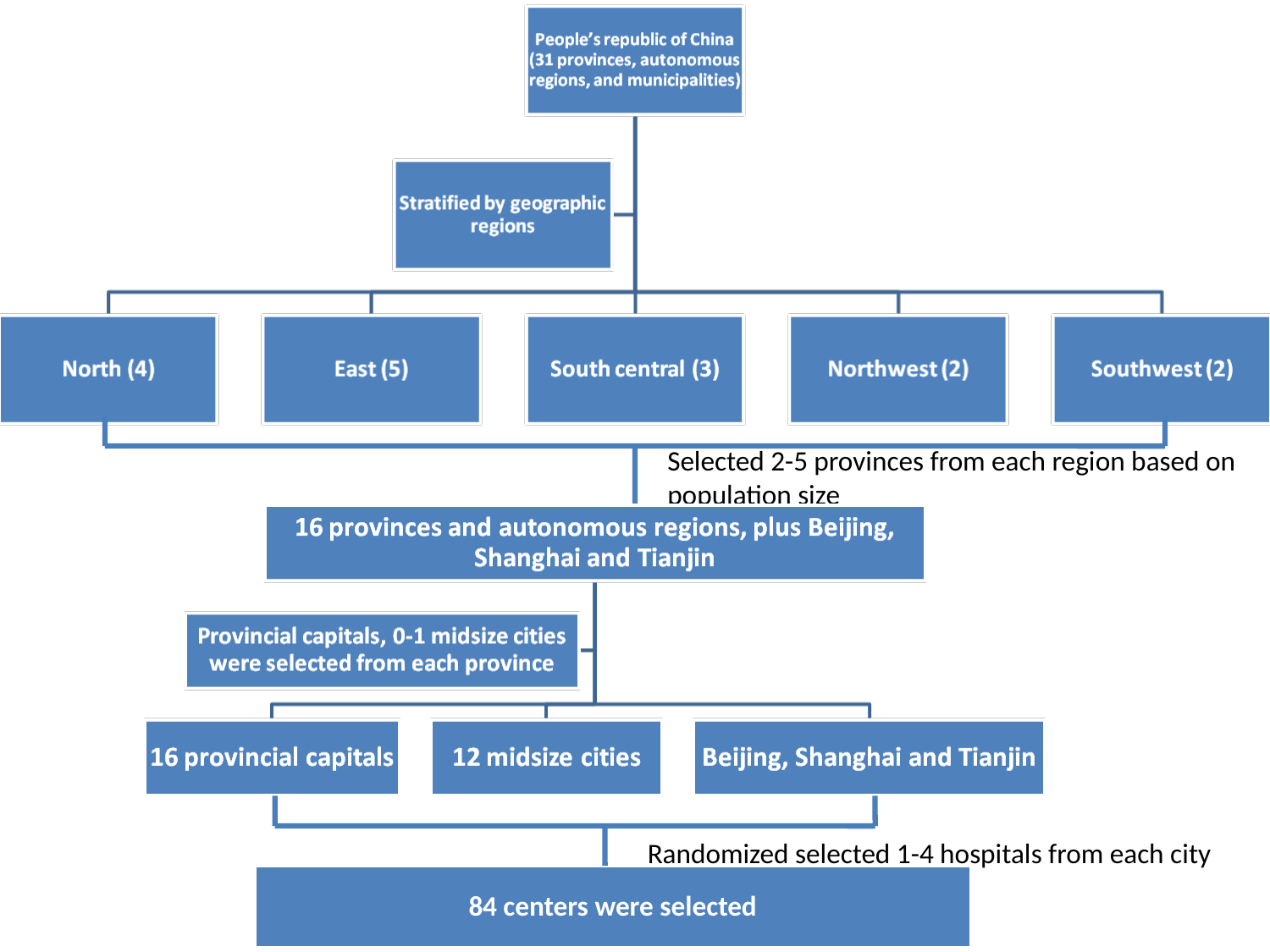

Selected 2-5 provinces from each region based on population size
Randomized selected 1-4 hospitals from each city
84 centers were selected
